# The Influence of Different Levels of Sodium Chloride, Sodium Nitrite, and Glucose on Biogenic Amines and Microbial Communities in Fermented Goat Meat Sausage

**DOI:** 10.3390/foods13060817

**Published:** 2024-03-07

**Authors:** Shuanghui Wu, Yin Niu, Jie Wang, Xiaofang Dao, Yaqiu Lin, Juan Chen

**Affiliations:** 1 College of Food Science and Technology, Southwest Minzu University, Chengdu 610225, China; sally595379141@163.com (S.W.);; 2 College of Animal and Veterinary Sciences, Southwest Minzu University, Chengdu 610041, China

**Keywords:** sodium chloride, sodium nitrite, glucose, biogenic amines, microbial diversity

## Abstract

The influence of different levels of sodium chloride, sodium nitrite, and glucose on the quality characteristics of spontaneously fermented goat meat sausage was investigated. The amounts of total biogenic amines in all the sausages ranged from 324.70 to 388.77 mg/kg; among them, spermine was the most abundant, with amounts ranging from 230.96 to 275.78 mg/kg. Increasing sodium chloride from 15 to 35 g/kg, the content of cadaverine, putrescine, tyramine, phenylethylamine, tryptamine, and total amines decreased, and Enterobacteriaceae counts decreased at the same time. Increasing sodium nitrite from 150 to 250 mg/kg, the content of cadaverine, histamine, and total amines decreased, while Enterobacteriaceae counts decreased simultaneously. Increasing glucose from 10 to 40 g/kg, the content of cadaverine, spermidine, and total amines decreased. *Enterococcus* was the most abundant genus across all the samples, and the relative abundance of *Enterococcus* was reduced obviously by increasing sodium nitrite and glucose levels. The top 10 differential bacterial taxa for each additive group were respectively obtained, and microbial biomarkers for each level of additive within its group were acquired, respectively. Through Pearson correlation, *Lactobacillus* was positively correlated with phenylethylamine, tryptamine, tyramine, and cadaverine, *Bacteroides* and *Sediminibacterium* were positively correlated with phenylethylamine and putrescine, respectively, suggesting they have the potential to produce biogenic amines. The results provided references for controlling the accumulation of biogenic amines in fermented goat meat sausage via the addition of auxiliary additives during the processing.

## 1. Introduction

Goat is an important source of meat, milk, yogurt, cheese, and other by-products, such as hide and skin [1]. Goat meat is high in protein, low in saturated fatty acids and cholesterol, and it is a healthier alternative compared to other red meats [2]. As one of the goat meat processed products, fermented goat meat sausage is produced around the world with various products and constitutes a cultural heritage in Europe, Asia, and Africa. Current research on goat meat sausage mainly focuses on the influence of processing technologies [3,4], starter cultures [5,6], and spices [6,7,8] on its physicochemical properties, flavor compounds, and biogenic amines.

Biogenic amines (BAs) are alkaline substances of low molecular weight. Although adequate levels of biogenic amines play a critical role in the normal physiological function of cells, consumption of foods with high levels of biogenic amines could lead to poisoning with symptoms including headache, vomiting, or diarrhea [9]. International regulations limiting biogenic amines vary from country to country. The United States Food and Drug Administration (FDA) requires histamine levels in fish products to be lower than 50 mg/kg [10]. And the European Union requires histamine levels in fish and fishery products not to exceed 100 mg/kg [11]. Studies have suggested biogenic amine content limits in food products of 100 mg/kg for histamine, 30 mg/kg for phenylethylamine, 100–800 mg/kg for tyramine [12], and 1000 mg/kg for the total biogenic amines [13]. Fermented sausage is suitable for the accumulation of biogenic amines due to its high nutrient content and microbiota diversity. In 40 Greek fermented sausage samples, the contents of putrescine and tyramine were determined to reach up to 492 mg/kg and 381 mg/kg, respectively. The content of histamine in 37% of all the samples was higher than 100 mg/kg [14]. An amount of 120 sausage samples collected from 10 different brands consumed in Van in Turkey were determined to be high in biogenic amine contents, such as from 0 to 1148.75 mg/kg for cadaverine, from 0 to 469.38 mg/kg for histamine, from 0 to 438.13 mg/kg for tyramine, from 0 to 554.375 mg/kg for spermidine, and from 0 to 614.38 mg/kg for spermine [15]. An amount of 50 artisanal and industrial dry fermented sausages in Serbia were subjected to an investigation of their biogenic amine profiles. Total biogenic amine content ranged from 37.3 to 1186 mg/kg, and the sum in 8% of the samples was determined to be very high (above 550 mg/kg). The content of histamine in one sample exceeded 100 mg/kg [9]. Therefore, it could be generally considered that the content of biogenic amines in fermented sausage is an important concern from a product safety perspective, and requires improvements in the hygiene of the production process.

In addition to the main material, the sausage processing needs some auxiliary materials to improve sensory characteristics and enhance the safety of the product, including commonly used spices and other additives [16]. Additives like sodium chloride, nitrite, and glucose could affect the growth and metabolism of decarboxylating microorganisms with the capability of producing biogenic amines, and then affect biogenic amine accumulation. Current research has focused on the effect of sodium chloride, sodium nitrite, and glucose on the production of biogenic amines; however, limited information is published on the influence of these additives on the microbiota community and associated biogenic amines in fermented sausages. The aim of this study is to investigate the differences in microbial communities and biogenic amine composition under different levels of additives (low, medium, and high addition levels of sodium chloride, sodium nitrite, and glucose) in spontaneously fermented goat meat sausage, and determine the relationship between the development of microorganisms and the formation of biogenic amines using chemical and high-throughput sequencing approaches. The results obtained could provide a better reference for controlling the accumulation of biogenic amines via the addition of auxiliary additives in fermented goat meat sausage.

## 2. Materials and Methods

### 2.1. Materials

Fresh lean meat and back fat of Jianzhou Big-Eared goat were provided by Sichuan Tian Di Yang Bioengineering Limited Corporation (Jianyang, China). Pig casings, sodium chloride, sodium nitrite, glucose, and sodium-L-ascorbate were purchased from local markets in Chengdu. Standard amines, containing 1,7-diaminoheptane, putrescine, tryptamine, histamine, tyramine, spermidine, spermine, and dansyl chloride were purchased from Shanghai Yuanye Bio-Technology Limited Corporation (Songjiang, Shanghai, China), cadaverine and phenylethylamine were purchased from Dr. Ehrenstorfer (Augsburg, Germany), methanol, acetonitrile, ammonium acetate, and acetic acid (all of them HPLC grade), were purchased from Thermo Fisher Scientific (Waltham, MA, USA). Other chemicals such as hydrochloric acid, diethyl ether, sodium bicarbonate, sodium hydroxide, and sodium chloride (all of them analytical grade) were purchased from Chengdu Kelong Chemical Corporation Limited (Chengdu, China).

### 2.2. Preparation of Fermented Goat Meat Sausages

The raw meat included 80% lean meat and 20% fat. After mincing, 5 g/kg of sodium L-ascorbate was added to the meat and mixed thoroughly. After that, the meat batter was divided into seven treatments, and then additions were made according to the design of Table 1. The ground meat with additives was maintained for 24 h at 0–4 °C in the refrigerator and then stuffed into natural pork casing (5.0 cm in diameter). Thereafter, all sausages were hung vertically outdoors and ripened for 14 days naturally without adding any starter cultures at temperature of 1–15 °C, and relative humidity of 33~80%.

Two independent batches of goat meat sausage were produced under the same condition. At the fourteenth day, six observations (triplicate observations for each batch) were collected and subjected to the following parameters: pH, moisture content, nitrite ion concentration, thiobarbituric acid reactive substances (TBARS), color, bacterial population, biogenic amine content. At the same time, six observations (triplicate observations for each batch) were collected and subjected to microbial diversity analyses.

### 2.3. Methods

#### 2.3.1. Physicochemical Properties Measurement

The pH value, moisture content, nitrite ion concentration, TBARS, and color measurement were measured according to the method of Chen et al. [17].

#### 2.3.2. Bacterial Populations Measurement

Ten grams of each sample were taken under sterile conditions. Total aerobic mesophilic counts were detected using Plate Count Agar (PCA) after incubation at 37 °C for 48 h. Presumptive lactic acid bacteria counts were determined on de Man Rogosa Sharpe Agar (MRSA) after incubation at 30 °C for 48 h. Staphylococci counts were measured on Mannitol Salt Agar (MSA) after incubation at 37 °C for 17 h. Enterobacteriaceae counts were detected using Violet Red Bile Glucose Agar (VRBGA) under incubation at 37 °C for 24 h. All above Agars were provided by Qing Dao Hope Bio-Technology Limited Corporation, Qingdao City, China.

#### 2.3.3. Biogenic Amines (BAs) Analysis

BAs in goat meat sausage samples were measured by high-performance liquid chromatography (HPLC, Agilent 1260, Agilent Technologies Co. Ltd., Palo Alto, CA, USA). Sample pretreatment, standard solution preparation, sample and standard derivatization, and determination were performed with reference to GB 5009.208-2016 (China) [18]. In brief, 10 g of the minced sausage was taken, and 500 μL of 1.0 mg/mL internal standard solution (1,7-diaminoheptane) was added. It was extracted twice with 20 mL of 5% (*w*/*w*) trichloroacetic acid solution; the supernatants were combined, and then a final volume of 50 mL was adjusted with 5% trichloroacetic acid. An amount of 10 mL of the extract was taken, 0.5 g sodium chloride and 10 mL n-hexane were added, and it was shaken for 5 min to remove fat from the extract. An amount of 5 mL of the defatted solution was taken, 5 mL of the mixed solution of chloroform and n-butanol (1:1) was added, and after shaking and centrifugation, the upper solution was extracted again, the extract was combined, and the final volume was adjusted to 10 mL with the mixed solution of chloroform and n-butanol (1:1). After that, 200 μL of 1 mol/L hydrochloric acid was added to 5 mL extract, then it was blow-dried under nitrogen in a water bath at 40 °C. After dissolving with 1 mL of 0.1 mol/L hydrochloric acid, the mixture was derivatized with dansyl chloride in alkaline medium. After removing excessive dansyl chloride, the extract was diluted with 1 mL of acetonitrile and then was filtered through 0.22 μm aperture filter. An amount of 20 μL of the above-mentioned filtrate was injected into an HPLC system, and the injection was repeated three times.

The column specifications, chromatographic conditions, mobile phase compositions, and gradient elution methods were in agreement with the study by Chen et al. [19].

#### 2.3.4. Microbial Diversity Analysis

##### Genomic DNA Extraction from the Samples

Samples of goat meat sausage were collected and stored in a refrigerator at −80 °C. The genomic DNA of the samples was extracted by MagPure Soil DNA LQ Kit (Magen Biotechnology Co., Ltd., Guangzhou, China), and then the concentration of DNA was detected by agarose gel electrophoresis and NanoDrop 2000 (Thermo Fisher Scientific, Waltham, MA, USA). Using genomic DNA as a template, universal primers (343F-5′-TACGGRAGGCAGCAG-3′ and 798R-5′-AGGGTATCTAATCCT-3′) were used to perform PCR on the V3-V4 region of microbial 16S rRNA using specific primers with barcode and Tks Gflex DNA Polymerase (Takara Bio Inc., Tokyo, Japan).

##### Library Construction

The PCR products were detected by electrophoresis, and then purified by AMPure XP beads (Beckman Coulter Life Sciences, West Sacramento, CA, USA). After purification, the PCR products were used as templates for the second round of PCR, and the second round of PCR amplification was carried out. The PCR products were quantified by Qubit dsDNA assay kit (Thermo Fisher Scientific, Waltham, MA, USA) after purification. Equal amounts of samples were mixed according to the concentration of PCR products and sequenced. Sequencing and analysis were conducted by OE Biotechnology Corporation Limited (Shanghai, China).

##### Bioinformatic Analysis

The sequencing results generate raw double-ended sequences, called raw data, in FASTQ format. The double-ended reads were then preprocessed using cutadapt software (2020.11) to detect and cut off the adapter. After trimming, low-quality sequences were filtered using DADA 2, and filtered according to QIIME 2 (2020.11) default parameters, noise reduction, splicing, and dechimeras, and representative sequences and Amplicon Sequence Variant (ASV) abundance tables were obtained. A sequence similarity of 100% was classified as one ASV unit. After selecting representative sequences for each ASV using the QIIME 2 software package, all representative sequences were aligned and annotated against the Silva database. The alpha diversity (Observed species, Good’s coverage, Chao1, Shannon, Simpson, and ACE index) was used to estimate the microbial diversity of the goat meat sausage samples, and species alignment was annotated using q^2^-feature-classifier with the default parameters.

### 2.4. Statistics Analysis

All the results were expressed as the means (of six observations) ± standard errors for each sample. All data were statistically analyzed using one-way ANOVA procedure of SPSS version software (IBM SPSS Statistics 22). Duncan’s multiple range test was used to determine significant differences between mean values, and *p*-values less than 0.05 were considered statistically significant.

## 3. Results and Discussion

### 3.1. Regular Physicochemical Properties of Goat Meat Sausages in Different Groups

The moisture contents of goat meat sausages in different groups are shown in Table 2. Within the sodium chloride group, the moisture content of T 2 (15 g/kg) was significantly lower than that of T 3 (35 g/kg) (*p* < 0.05), and the higher the addition of sodium chloride, the higher the moisture content. The reason might be that sodium chloride increases the ionic strength of meat, and then a large quantity of myofibrillar protein is dissolved to trap water through the combination of protein with sodium and chloride ions, so that the water retention of meat is improved [20]. Within the sodium nitrite group, the moisture content of T 5 (250 mg/kg) was significantly lower than that of T 4 (200 mg/kg). The pH of T 1 (10 g/kg) was significantly higher than that of T 6 (25 g/kg) and T 7 (40 g/kg) (*p* < 0.05), suggesting that increasing glucose level may lead to a decrease in the pH of the samples. The nitrite ion content of all the samples (3.06 to 8.42 mg/kg) was lower than 30 mg/kg, which was complied with the residue limitation of nitrite ions in fermented meat products in China [21]. Notably, the higher sodium nitrite treatments resulted in obviously higher nitrite ion concentration in sausages. Although the combination of nitrites and biogenic amines caused the formation of nitrosamines with potential carcinogenic effects, other factors are also effective in the formation of nitrosamines such as processing methods, processing temperature, and time [22], and the addition of sodium L-ascorbate in the formulation of sausage could provide a protective function against nitrosamine formation. The TBARS value of all the samples (0.543 to 0.762 mg MDA/kg) was considered to be acceptable according to the limit of 2–2.5 mg MDA/kg meat products [23]. Sausage color was determined by a combination of lightness (L*), redness (a*), and yellowness (b*). The L* value for goat meat sausage decreased gradually with the increasing sodium chloride content. With respect to the a* and b* values, the type and amount of additives had no significant effect on them (*p* > 0.05).

### 3.2. Regular Microbiological Populations of Goat Meat Sausages in Different Groups

The aerobic mesophilic counts, presumptive lactic acid bacteria counts, presumptive staphylococci counts, and Enterobacteriaceae counts of goat meat sausage samples are shown in Table 3. The count of presumptive staphylococci in T 3 was significantly (*p* < 0.05) higher than that of T 1 and T 2, and the higher the concentration of sodium chloride, the higher the counts of presumptive staphylococci in the samples. The counts of presumptive lactic acid bacteria in T 2 and T 3 were significantly higher than those of T 1. The counts of Enterobacteriaceae in T 3 and T 1 were significantly lower than those of T 2, and the higher the concentration of sodium chloride, the lower the counts of Enterobacteriaceae in the samples. Among the sodium nitrite group, the counts of presumptive staphylococci in T 1 were significantly (*p* < 0.05) higher than that of T 5 and there were no significant differences between T 5 and T 4. The counts of presumptive lactic acid bacteria were significantly (*p* < 0.05) higher in T 4 than those of T 1 and T 5. The counts of Enterobacteriaceae in T 4 and 5 were significantly (*p* < 0.05) lower than those of T 1, and the higher the addition of sodium nitrite, the lower the Enterobacteriaceae counts of goat meat sausage samples. Similar results have been reported by Hospital and colleagues. It was found that a gradual increase in nitrate and nitrite concentration (from 0 to 150 mg/kg, separately) brought out a gradual decrease in Enterobacteriaceae counts during the ripening of chorizo [24]. Within the glucose group, the count of presumptive staphylococci was significantly (*p* < 0.05) higher in T 7 than that of T 6 and T 1. The count of presumptive lactic acid bacteria in T 6 was significantly (*p* < 0.05) higher than that of T 7, which was then significantly higher than that of T 1. The Enterobacteriaceae counts in T 7 and T 6 were significantly (*p* < 0.05) lower than those of T 1. In industrial formulations, especially in fermented sausages, sugars such as glucose, sucrose, and lactose are added in order to raise the counts of lactic acid bacteria and then improve the LAB fermentation process. González-Fernández and colleagues found that lactic acid bacteria counts in chorizo dry sausage were significantly higher in the 1% glucose addition batch than that of the 0.1% glucose addition batch [25]. A similar phenomenon was observed in our study. Lactic acid bacteria counts were significantly higher in elevated glucose treatments; both T 6 and T 7 were higher in comparison to the control T 1. However, Enterobacteriaceae counts were largely decreased as a consequence of the elevated glucose level. Therefore, in elevated glucose treatments, the total aerobic mesophilic counts did not show an increase, but a slight decrease compared to the control.

### 3.3. Biogenic Amine Contents of Goat Meat Sausages in Different Groups

The biogenic amine contents of fermented goat meat sausages with different additives are shown in Table 4. The total amounts of BAs in all the samples ranged from 324.70 to 388.77 mg/kg, lower than the maximum limit of 1000 mg/kg for food [13]. Spermine was the most abundant biogenic amine for goat meat sausages, with contents ranging from 230.96 to 275.78 mg/kg. Similar results were observed in the study of Çiçek & Tokatli. In their study, the highest content of biogenic amine in the Turkish fermented beef sausages Bez sucuk was spermine throughout the whole processing period (14 days), and the spermine content from different treatment groups (90:10, 80:20, and 70:30 of beef to fat ratios) approached 343.34, 306.95, and 321.63 mg/kg, respectively [26]. The content of histamine in all the samples ranged from 7.87 mg/kg to 12.90 mg/kg, below the legal limit of 100 mg/kg for food products [12]. The content of tyramine in all the samples ranged from 46.86 to 56.36 mg/kg, which was below the threshold value of 100–800 mg/kg for food products [12]. The content of phenylethylamine in all the samples ranged from 5.46 to 11.37 mg/kg, under the legal limit of 30 mg/kg for food products [12]. Other biogenic amines included 3.25 to 12.80 mg/kg for tryptamine, 8.20 to 10.31 mg/kg for putrescine, and 0.8 to 3.79 mg/kg for cadaverine, respectively.

Larger amounts of tyramine and cadaverine were detected in Portuguese traditional sausages processed with a low salt amount of 3%, in comparison to sausages processed with a high salt amount of 6% [27]. The research group directed by Gardini et al. investigated the influence of technological factors on the growth of microorganisms and the production of biogenic amines during the whole processing of the dry fermented sausage Salame Veronese inoculated with the tyraminogenic strain *E. faecalis* EF37. They found that increasing sodium chloride from 0% to 5% led to lower counts of Enterobacteriaceae and simultaneously reduced the accumulation of tyramine and phenylethylamine [28]. The high addition of NaCl was largely demonstrated to be able to inhibit the multiplication of microorganisms with the capability of producing biogenic amines, such as *Lactobacillus*, *Bacillus*, *Pseudomonas* and *Enterococcus*, and prevent the production of biogenic amines in foods [29]. Similarly, in this study with the increase in the sodium chloride addition (15, 25, 35 mg/kg), the content of cadaverine, putrescine, tyramine, phenylethylamine, and tryptamine gradually decreased, resulting in a decreasing trend of total biogenic amines; meanwhile, the counts of Enterobacteriaceae decreased.

Moreover, Gençcelep et al. found that in a traditional Turkish dry fermented sausage, Sucuk, the addition of nitrite in amounts of 75 and 150 mg/kg significantly prevented the accumulation of putrescine and tyramine compared to no sodium nitrite addition in sausage without starter cultures, and the counts of Enterobacteriaceae fell below detectable levels rapidly across all the treatments [30]. The addition of nitrite in amounts of 100 and 200 mg/kg led to decreased levels of 2-phenylethylamine, tryptamine, putrescine, cadaverine, tyramine, and histamine in Sucuk produced with starter cultures [31]. Similar results were described in our results. With the increase in the sodium nitrite addition (150, 200, 250 mg/kg), the content of cadaverine, histamine, and total amines in goat meat sausages showed a decreasing trend; also, the counts of Enterobacteriaceae showed a descending trend.

Furthermore, in the Italian salami abruzzese, an increased amount of added sugar in strategy F 1 (1.9 g/kg glucose + 1.9 g/kg sucrose) and strategy F 2 (3 g/kg glucose + 2 g/kg sucrose) reduced the accumulation of cadaverine by 43% and 21%, respectively, compared to the control manufactured with 2.6 g/kg of sugar [32]. In the present study, with the increase in the glucose addition (10, 25, 40 g/kg), the content of cadaverine, spermidine, and total amines tended to decrease. Additionally, the counts of Enterobacteriaceae in the 25 and 40 g/kg glucose treatments were significantly reduced in contrast to that of the 10 g/kg glucose treatment.

### 3.4. Bacterial Diversity of Goat Meat Sausages in Different Groups

#### 3.4.1. Sequencing Data Statistics and ASV Classification

After clipping, quality filtering, splicing, and de-chimerisation of the original double-ended sequences from the off-machine, statistics were performed. There were a total of 42 samples of goat meat sausage. The number of sequences obtained after noise reduction and splicing ranged from 64,962 to 74,262, and the number of sequences obtained after chimera removal ranged from 48,683 to 71,718. The total number of ASVs from 42 samples was 4,147. The number of ASVs in each sample ranged from 94 to 831.

**Figure 1:**
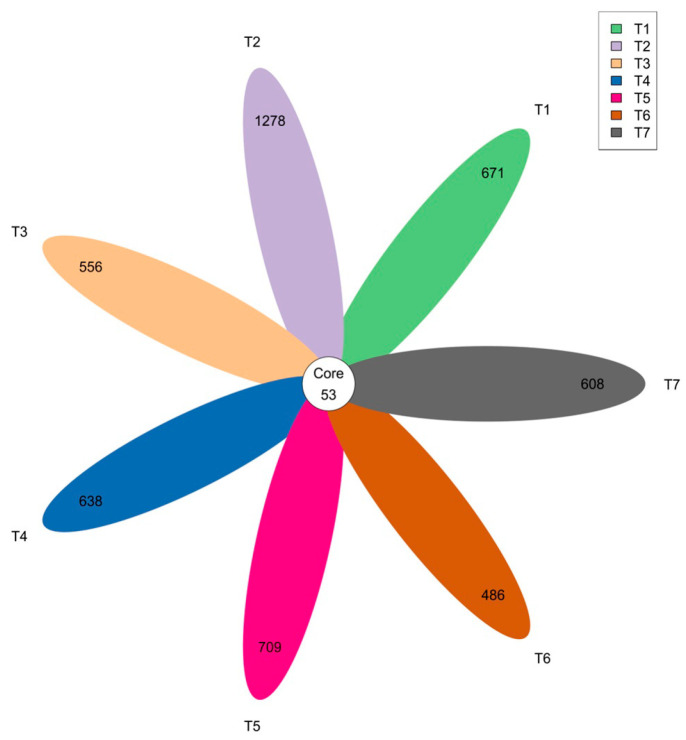
A Venn diagram of the microbial community of fermented goat meat sausages in different treatments and groups.

To simplify the workload of species annotation, ASVs were obtained by clustering according to the requirement of 100% sequence similarity, and then the number of common and unique ASVs between different groups was analyzed, as shown in Figure 1. In total, 724, 1,331, 609, 691, 762, 539, and 661 ASVs were obtained from T 1, 2, 3, 4, 5, 6, and 7, respectively. Overall, only 53 ASVs were shared by all the samples, indicating that bacterial diversity between samples was not highly similar. Therefore, it could be concluded that the richness and diversity of microbial composition varied largely across treatments and groups.

#### 3.4.2. Alpha Diversity Analysis

The microbiota diversity of goat meat sausages in different groups is presented in Table 5. Good’s coverage was 1.00 for all the samples, indicating that almost all of the bacterial phylotypes of sausage samples were captured. Observed species ranged from 120.60 to 258.53. The Chao 1 and ACE indices gradually decreased with the increase in the sodium chloride addition, indicating that the community richness decreased with sodium chloride increasing. It could be inferred that the high concentration of sodium chloride would inhibit the growth and multiplication of certain kinds of microorganisms.

#### 3.4.3. Bacterial Community Analysis

In order to investigate the bacterial community of fermented goat meat sausage in different groups, bacterial 16S rRNA gene sequences were classified at the phylum and genus levels. A total of 22 phyla and 406 genera were detected.

**Figure 2:**
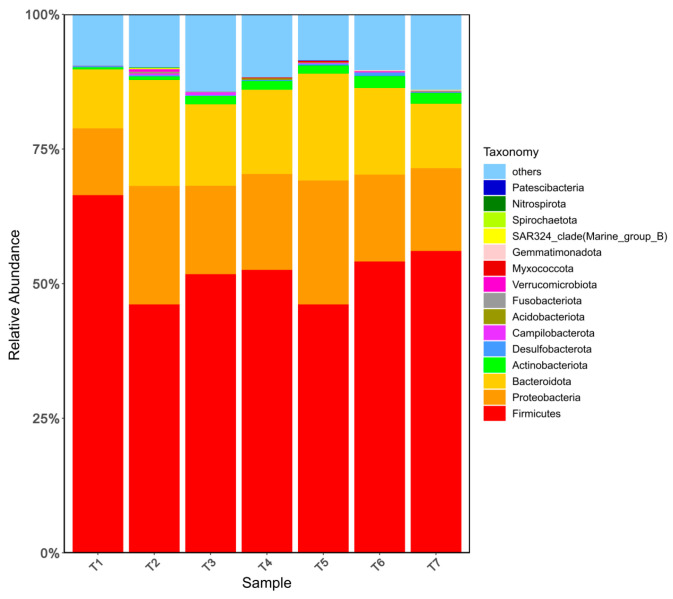
Relative abundances of bacteria at the phylum level of fermented goat meat sausage in different treatments and groups (the top 15 phyla).

**Figure 3:**
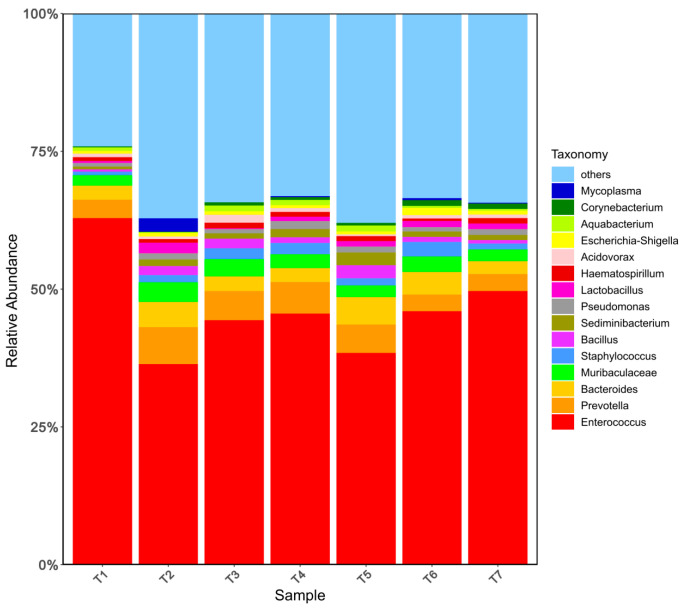
Relative abundances of bacteria at the genus level of fermented goat meat sausage in different treatments and groups (the top 15 genera).

The relative abundance of the top 15 bacteria at the phylum level in goat meat sausages is displayed in Figure 2. *Firmicutes*, *Proteobacteria*, and *Bacteroidota* were the predominant phyla and their relative abundances were higher than 10%. *Firmicutes* was recognized as the most predominant phylum with a relative abundance of over 46.13%, followed by *Proteobacteria* with a relative abundance of nearly 12.40%, and *Bacteroidota* with a relative abundance of over 10.98%. Similar results were observed by Wang et al. They found that *Firmicutes* and *Proteobacteria* were the dominant phyla with a total proportion of above 90% at all fermentation stages (fermenting 0, 10, 20, 30 days) of Chinese Cantonese sausage [33]. In Figure 3, an analysis of the microbial community at the genus level was performed, and the top 15 genera of microbiota in all the samples were presented. *Enterococcus* was the most abundant genus, with the relative abundance ranging from 36.38% to 62.88%. Following that, *Prevotella* and *Bacteroides* had the relative abundance ranging from 3.03% to 6.71%, and from 2.35% to 4.61%, respectively. In comparison to T 1 with the lowest level of sodium nitrite (150 mg/kg) and glucose (10 g/kg), the relative abundance of *Enterococcus* was reduced obviously by the elevating levels of the two additives, respectively. Although this image was not fully observed in the sodium chloride group, the relative abundance of *Enterococcus* was also reduced by increasing sodium chloride from 25 g/kg to 35 g/kg.

#### 3.4.4. Differential Bacterial Taxa Analysis

**Figure 4:**
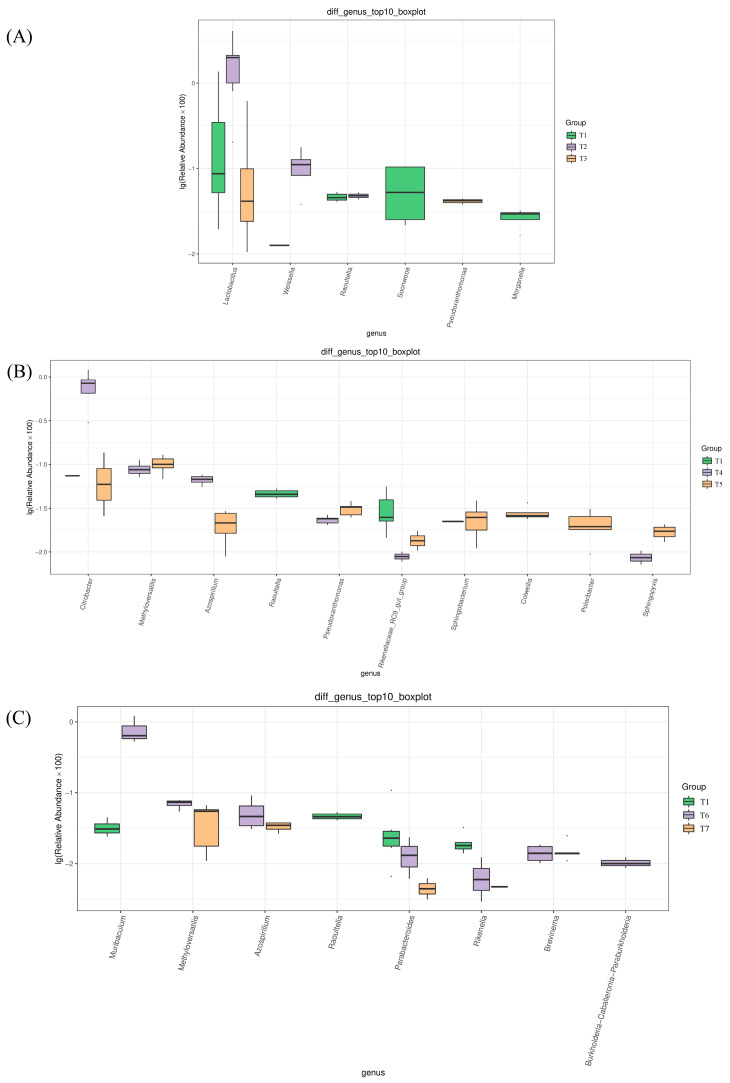
Analysis of differences in bacteria of fermented goat meat sausages at the genus level in different groups through the ANOVA analysis. (**A**) Sodium chloride group, (**B**) sodium nitrite group, and (**C**) glucose group.

The ANOVA was used to analyze the significantly differential bacterial taxa, and statistical analysis was performed at the genus level. Differential bacterial taxa were first screened according to *p* < 0.05, then the top 10 relative abundance genera were selected. In Figure 4A, the top 10 differential bacterial taxa in goat meat sausages within the sodium chloride group were *Lactobacillus*, *Weissella*, *Raoultella*, *Soonwooa*, *Pseudoxanthomonas*, and *Morganella*. The top 10 differential bacterial taxa within the sodium nitrite group were *Citrobacter*, *Methyloversatilis*, *Azospirillum*, *Raoultella*, *Pseudoxanthomonas*, *Rikenellaceae* RC9 gut group, *Sphingobacterium*, *Colwellia*, *Polaribacter*, and *Sphingopyxis* (Figure 4B). The top 10 differential bacterial taxa in goat meat sausages within the glucose group were *Muribaculum*, *Methyloversatilis*, *Azospirillum*, *Raoultella*, *Parabacteroides*, *Rikenella*, *Brevinema*, and *Paraburkholderia* (Figure 4C).

In addition, linear discriminant analysis coupled with effect size measurements (LEfSe) was performed to obtain the greatest differences in taxa within the samples. A total of 20 bacterial clades consisting of 1 phylum, 1 order, 6 families, and 12 genera were considered to be the differential taxa within the sodium chloride group and are shown in Supplementary Figure S1A,B. *Lactobacillus*, *Aeromonas*, and *Weissella* were biomarkers significantly enriched in T 2 with the lowest level of 15 g/kg sodium chloride, and *Pseudoxanthomonas* was the biomarker of T 3 with the highest addition of 35 g/kg sodium chloride. From Supplementary Figure S1C,D, it is shown that a total of 40 bacterial clades were observed for the sodium nitrite group, consisting of 1 phyla, 3 orders, 13 families, and 22 genera. The genera of *Luteimonas*, *Rikenellaceae* RC9 gut group, *Ruminococcus*, *Soonwooa*, *Raoultella*, *Parasutterella*, and *Barnesiella* were the biomarkers that distinguished the lowest level of 150 mg/kg sodium nitrite in T 1 from the other two treatments. *Stenotrophomonas, Hyphomicrobium*, *Hydrogenophaga*, *Azospirillum*, and *Burkholderia Caballeronia Paraburkholderia* were the biomarkers significantly enriched in T 4 (200 mg/kg sodium nitrite), and *Prevotellaceae UCG 001, Rikenella*, *Alistipes*, *Methyloversatilis*, *Terrinonas*, *Pseudoxanthomonas*, *Colwellia, Polaribacter, Silanimonas, and Sphingobacterium* were the biomarkers of T 5 with the highest level of 250 mg/kg sodium nitrite. A total of 23 bacterial clades were observed for the glucose group (as shown in Supplementary Figure S1E,F), including 1 order, 7 families, and 15 genera. *Rikenella*, *Parabacteroides*, *Pseudoalteromonas*, *Raoultella*, *Allobaculum*, *Alloprevotella*, *Klebsiella*, *Parasutterella*, and *Barnesiella* were the biomarkers that distinguished T 1 with the lowest addition of 10 g/kg glucose from the other two treatments. *Burkholderia_Caballeronia_Paraburkholderia*, *Azospirillum*, *Desulfovibrio*, *Methyloversatilis*, and *Mycoplasma* were the biomarkers significantly enriched in T 6 (25 g/kg glucose), and the biomarker of T 7 with the highest addition of 40 g/kg glucose was *Pseudoxanthomonas*.

### 3.5. Correlations between Bacterial Genera and Biogenic Amines

**Figure 5:**
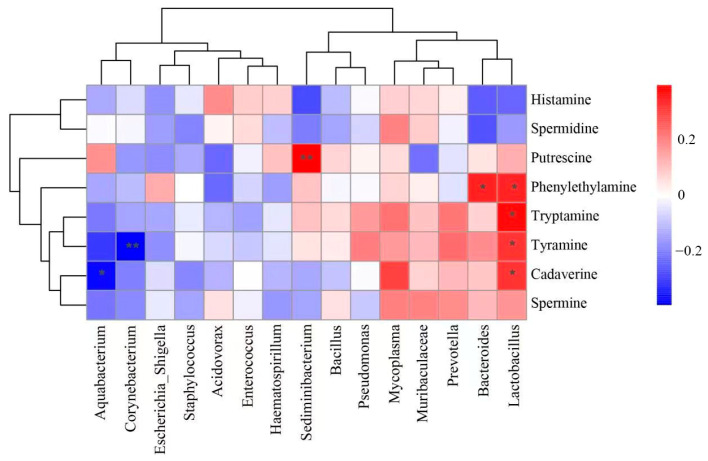
Correlation heatmap of bacterial relative abundance and biogenic amine content * 0.01 < *p* ≤ 0.05, ** 0.001 < *p* ≤ 0.01.

In this study, the correlation between biogenic amines and the top 15 genera in the goat meat sausage samples was evaluated using the Pearson correlation and is illustrated with a heatmap in Figure 5. The relative abundances of five genera showed significant correlations with the contents of certain biogenic amines (*p* < 0.05). The relative abundance of *Lactobacillus* had significant positive correlations with the content of phenylethylamine, tryptamine, tyramine, and cadaverine, respectively (*p* < 0.05). *Bacteroides* was positively correlated with phenylethylamine (*p* < 0.05), and *Sediminibacterium* was positively correlated with putrescine (*p* < 0.01), respectively. *Aquabacterium* had a significant negative correlation with cadaverine (*p* < 0.05), and *Corynebacterium* had an extremely significant negative correlation with tyramine (*p* < 0.01).

Fermented sausage is an open microbial fermentation, and it is generally regarded that the formation of biogenic amines of fermented sausages is closely related to the development of the microbiota-producing amino acid decarboxylase. Lactic acid bacteria in fermented food can accumulate biogenic amines and the main LAB genera such as *Lactobacillus*, *Enterococci*, and so on have been associated with high levels of amine compounds [34]. Under the addition of different plant extracts in fermented sausages, *Lactobacillus* and *Pseudomonas* showed significantly positive correlations with histamine, tryptamine, and cadaverine, respectively, indicating the promotion of biogenic amine accumulation by the two genera [35]. Besides, the decarboxylating capabilities of bacteria in fermented meat products were investigated. For example, among 329 bacterial isolates from Swiss fermented sausages, 66 coagulase-negative Staphylococci and 54 *Enterococci* were identified to be decarboxylating strains and produce abundant tyramine [36]. The decarboxylase activity of 76 bacterial strains isolated from European traditional fermented sausages was investigated and the results showed that 22 lactic acid bacteria and 4 coagulase-negative Staphylococci could decarboxylate one or more amino acids [32]. In the present study, with the variation of sodium chloride, sodium nitrite, and glucose added to goat meat sausage, *Lactobacillus* showed significant positive correlations with phenylethylamine, tryptamine, tyramine, and cadaverine, and *Bacteroides* showed significant positive correlation with phenylethylamine. Both *Enterococcus* and *Pseudomonas* did not present significant correlations with any biogenic amines. It could be seen that the same species or genera bacterium may have different biogenic amine production performances. Therefore, it was proposed that the accumulation of biogenic amines in fermented meat products seemed to be more strain-dependent than species or genera-specific [34]. Free amino acid status in different matrices might be another important factor influencing the production of biogenic amines by microorganisms [37].

## 4. Conclusions

Under different levels of sodium chloride (15, 25, 35 g/kg), sodium nitrite (150, 200, 250 mg/kg), and glucose (10, 25, 40 g/kg), the influence of these additives on regular physicochemical properties, microbiological populations, biogenic amine formation, and bacterial diversity in spontaneously fermented goat meat sausage were revealed.

In the range of 15~35 g/kg, the higher the addition of sodium chloride, the higher the moisture content of the goat meat sausage. Increasing glucose levels from 10 to 40 g/kg led to a decrease in the pH of the samples. The nitrite ion content of all the goat meat sausages (3.06 to 8.42 mg/kg) was lower than 30 mg/kg. The TBARS value of all the goat meat sausages (0.54 to 0.76 mg MDA/kg) was considered to be acceptable. The amounts of total biogenic amines in all the goat meat samples were lower than 1000 mg/kg, ranging from 324.70 to 388.77 mg/kg; among them, spermine was the most abundant one, ranging from 230.96 to 275.78 mg/kg. Based on regular microbiological populations and biogenic amines analysis, after increasing the sodium chloride from 15 to 35 g/kg, the content of cadaverine, putrescine, tyramine, phenylethylamine, tryptamine, and total amines decreased, and Enterobacteriaceae counts decreased at the same time. Increasing sodium nitrite from 150 to 250 mg/kg, the content of cadaverine, histamine, and total amines decreased, while Enterobacteriaceae counts decreased simultaneously. Increasing glucose from 10 to 40 g/kg, the content of cadaverine, spermidine, and total amines decreased. Through bacteria community analysis, *Enterococcus* was determined to be the most abundant genus across all the samples, with the relative abundance ranging from 36.38% to 62.88%. And the relative abundance of *Enterococcus* was reduced obviously by increasing the sodium nitrite and glucose levels. The top 10 differential bacterial taxa in goat meat sausages within each additive group were respectively obtained through ANOVA analysis. Furthermore, microbial biomarkers for each level of additive within its respective group were acquired using LEfSe. The relative abundances of the three genera showed significant positive correlations with the contents of certain biogenic amines through Pearson correlation. *Lactobacillus* was positively correlated with phenylethylamine, tryptamine, tyramine, and cadaverine (*p* < 0.05); *Bacteroides* was positively correlated with phenylethylamine (*p* < 0.05) and *Sediminibacterium* was positively correlated with putrescine (*p* < 0.01), respectively.

## Figures and Tables

**Table 1:** Design of experimental groups.

Additives	T 1 (Control)	Sodium Chloride Group		Sodium Nitrite Group		Glucose Group	
		T 2	T 3	T 4	T 5	T 6	T 7
Sodium chloride	25 g/kg	15 g/kg	35 g/kg	25 g/kg	25 g/kg	25 g/kg	25 g/kg
Sodium nitrite	150 mg/kg	150 mg/kg	150 mg/kg	200 mg/kg	250 mg/kg	150 mg/kg	150 mg/kg
Glucose	10 g/kg	10 g/kg	10 g/kg	10 g/kg	10 g/kg	25 g/kg	40 g/kg

T is the abbreviation of treatment.

**Table 2:** Regular physicochemical properties of fermented goat meat sausages in different groups.

Properties	T 1 (Control)	Sodium Chloride Group		Sodium Nitrite Group		Glucose Group	
		T 2	T 3	T 4	T 5	T 6	T 7
Moisture content	17.81 ± 0.78 ^bc^	15.11 ± 0.42 ^ab^	19.73 ± 0.89 ^c^	20.67 ± 1.32 ^c^	12.74 ± 0.68 ^a^	16.06 ± 1.27 ^b^	17.79 ± 0.85 ^bc^
pH value	5.90 ± 0.01 ^cd^	5.85 ± 0.01 ^b^	5.87 ± 0.01 ^bc^	5.88 ± 0.01 ^bcd^	5.92 ± 0.01 ^d^	5.76 ± 0.02 ^a^	5.78 ± 0.03 ^a^
Nitrite ion concentration	4.06 ± 0.12 ^b^	3.74 ± 0.13 ^ab^	3.62 ± 0.19 ^ab^	7.79 ± 0.44 ^c^	8.42 ± 0.17 ^c^	3.27 ± 0.16 ^a^	3.06 ± 0.18 ^a^
TBARS	0.64 ± 0.01 ^bc^	0.59 ± 0.02 ^ab^	0.54 ± 0.02 ^a^	0.64 ± 0.02 ^bc^	0.65 ± 0.03 ^bc^	0.76 ± 0.01 ^d^	0.68 ± 0.03 ^c^
L*	37.38 ± 1.71 ^ab^	39.27 ± 2.77 ^b^	36.30 ± 0.97 ^ab^	37.91 ± 2.50 ^ab^	34.16 ± 1.63 ^ab^	36.00 ± 1.28 ^ab^	32.43 ± 1.73 ^a^
a*	12.14 ± 2.20 ^b^	12.20 ± 0.31 ^b^	12.30 ± 0.69 ^b^	8.86 ± 0.63 ^a^	12.65 ± 0.43 ^b^	11.70 ± 0.67 ^b^	13.31 ± 0.48 ^b^
b*	13.95 ± 0.81 ^a^	14.38 ± 1.10 ^a^	14.80 ± 0.42 ^a^	12.73 ± 1.09 ^a^	14.17 ± 1.26 ^a^	13.36 ± 0.67 ^a^	13.82 ± 0.86 ^a^

^a,b,c,d^ within each row means with different letters are significantly different (*p* < 0.05). Unit for moisture content is %, unit for nitrite ion concentration is mg/kg, unit for TBARS is mg MDA/kg.

**Table 3:** Regular microbiological populations of fermented goat meat sausages in different groups.

Microbiological Populations	T 1 (Control)	Sodium Chloride Group		Sodium Nitrite Group		Glucose Group	
		T 2	T 3	T 4	T 5	T 6	T 7
Total aerobic mesophilic count	6.95 ± 0.04 ^c^	6.13 ± 0.05 ^a^	6.39 ± 0.03 ^b^	6.42 ± 0.04 ^b^	5.99 ± 0.01 ^a^	6.54 ± 0.04 ^b^	6.39 ± 0.06 ^b^
Presumptive lactic acid bacteria	5.97 ± 0.02 ^b^	6.32 ± 0.01 ^d^	6.23 ± 0.04 ^d^	6.20 ± 0.02 ^cd^	5.56 ± 0.03 ^a^	6.80 ± 0.01 ^e^	6.10 ± 0.01 ^c^
Presumptive staphylococci	5.97 ± 0.02 ^cd^	5.50 ± 0.07 ^a^	6.12 ± 0.01 ^de^	5.80 ± 0.01 ^b^	5.82 ± 0.02 ^bc^	5.99 ± 0.01 ^d^	6.25 ± 0.03 ^e^
Enterobacteriaceae	4.60 ± 0.02 ^d^	4.76 ± 0.03 ^e^	4.45 ± 0.03 ^c^	4.05 ± 0.02 ^b^	3.78 ± 0.04 ^a^	3.71 ± 0.04 ^a^	3.95 ± 0.04 ^b^

^a,b,c,d,e^ within each row means with different letters are significantly different (*p* < 0.05). Unit is lg CFU/g.

**Table 4:** Biogenic amines of fermented goat meat sausages in different groups (mg/kg).

Biogenic Amines	T1 (Control)	Sodium Chloride Group		Sodium Nitrite Group		Glucose Group	
		T 2	T 3	T 4	T 5	T 6	T 7
Tryptamine	3.87 ± 0.23 ^a^	12.80 ± 0.66 ^d^	3.25 ± 0.08 ^a^	10.41 ± 0.29 ^c^	5.91 ± 0.33 ^b^	4.10 ± 0.14 ^a^	6.01 ± 0.32 ^b^
Phenylethylamine	8.75 ± 0.25 ^b^	10.20 ± 0.50 ^cd^	5.46 ± 0.20 ^a^	8.37 ± 0.57 ^b^	10.05 ± 0.35 ^c^	11.37 ± 0.30 ^d^	8.46 ± 0.24 ^b^
Putrescine	8.67 ± 0.36 ^a^	9.16 ± 0.22 ^ab^	8.20 ± 0.15 ^a^	9.83 ± 0.56 ^bc^	10.31 ± 0.19 ^c^	8.42 ± 0.15 ^a^	9.01 ± 0.18 ^ab^
Cadaverine	2.60 ± 0.08 ^d^	3.79 ± 0.10 ^e^	0.80 ± 0.06 ^a^	1.41 ± 0.12 ^b^	0.95 ± 0.03 ^a^	1.78 ± 0.16 ^c^	1.38 ± 0.13 ^b^
Histamine	11.19 ± 0.78 ^b^	11.07 ± 0.14 ^b^	12.90 ± 0.23 ^c^	10.55 ± 0.43 ^b^	7.87 ± 0.26 a	8.48 ± 0.12 ^a^	11.60 ± 0.19 ^b^
Tyramine	49.54 ± 0.27 ^ab^	56.36 ± 1.64 ^d^	46.86 ± 0.51 ^a^	52.59 ± 0.73 ^c^	51.39 ± 0.44 ^bc^	49.40 ± 1.01 ^ab^	47.05 ± 0.82 ^a^
Spermidine	10.56 ± 1.21 ^ab^	9.59 ± 0.87 ^ab^	10.59 ± 0.84 ^ab^	11.78 ± 0.33 ^b^	8.24 ± 0.53 ^a^	9.84 ± 0.92 ^ab^	10.24 ± 0.12 ^ab^
Spermine	255.25 ± 3.28 ^b^	275.78 ± 2.05 ^c^	256.56 ± 2.79 ^b^	231.36 ± 1.25 ^a^	238.4 ± 0.92 ^ab^	243.09 ± 0.49 ^ab^	230.96 ± 1.55 ^a^
Total amine	350.42 ± 4.74 ^b^	388.77 ± 3.21 ^c^	344.62 ± 2.83 ^ab^	336.29 ± 1.52 ab	333.12 ± 1.58 ab	336.49 ± 2.15 ^ab^	324.70 ± 2.40 ^a^

^a,b,c,d,e^ within each row means with different letters are significantly different (*p* < 0.05).

**Table 5:** Alpha diversity indices of fermented goat meat sausages in different groups.

Groups	T 1 (Control)	Sodium Chloride Group		Sodium Nitrite Group		Glucose Group	
		T 2	T 3	T 4	T 5	T 6	T 7
Chao1	173.04	258.82	138.51	162.10	178.91	121.19	141.20
Goods coverage	1.00	1.00	1.00	1.00	1.00	1.00	1.00
Shannon	3.13	5.03	4.08	4.21	4.72	4.07	3.86
Observed species	171.90	258.53	138.05	161.28	178.50	120.60	140.42
Simpson	0.59	0.83	0.76	0.76	0.81	0.75	0.73
ACE	171.78	259.07	138.84	164.39	178.72	122.24	141.14

## Data Availability

The original contributions presented in the study are included in the article/supplementary material, further inquiries can be directed to the corresponding authors.

## Supplementary Materials

The following supporting information can be downloaded at: https://www.mdpi.com/article/10.3390/foods13060817/s1, Figure S1: Taxonomic differences of bacteria community of fermented goat meat sausages in different groups obtained from the LEfSe analysis. (A), (C), and (E) represent taxonomic cladograms for sodium chloride group, sodium nitrite group, and glucose group, respectively; (B), (D) and (F) indicate LDA scores distribution bars for sodium chloride group, sodium nitrite group and glucose group, respectively. LDA score is over 2. Labels beginning with o_, order; f_, family; g_, genus.
