# Supplementary material for: The Influence of Different Levels of Sodium Chloride, Sodium Nitrite, and Glucose on Biogenic Amines and Microbial Communities in Fermented Goat Meat Sausage
Source: Foods. 2024 Mar 7;13(6):817. doi: 10.3390/foods13060817 (PMC10969002; doi:10.3390/foods13060817)
Supplement: Supplementary file 1 [file foods-13-00817-s001.zip › foods-2885800-supplementary.pdf]

Supplementary Figure

Figure S1. Taxonomic differences of bacteria community of fermented goat meat sausages in different groups obtained from the LEfSe analysis. (A), (C), and (E) represent taxonomic cladograms for sodium chloride group, sodium nitrite group, and glucose group, respectively; (B), (D) and (F) indicate LDA scores distribution bars for sodium chloride group, sodium nitrite group and glucose group, respectively. LDA score is over 2. Labels beginning with o_, order; f_, family; g_, genus.

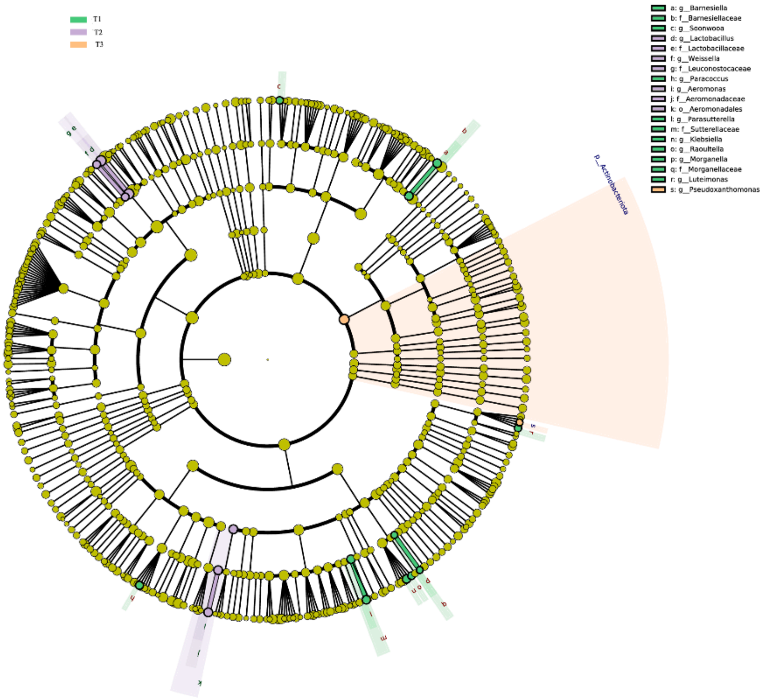

(A)

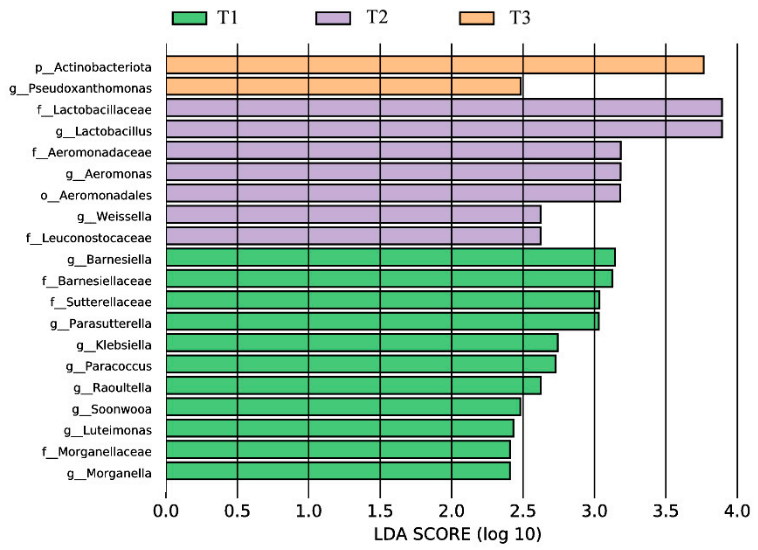

(B)

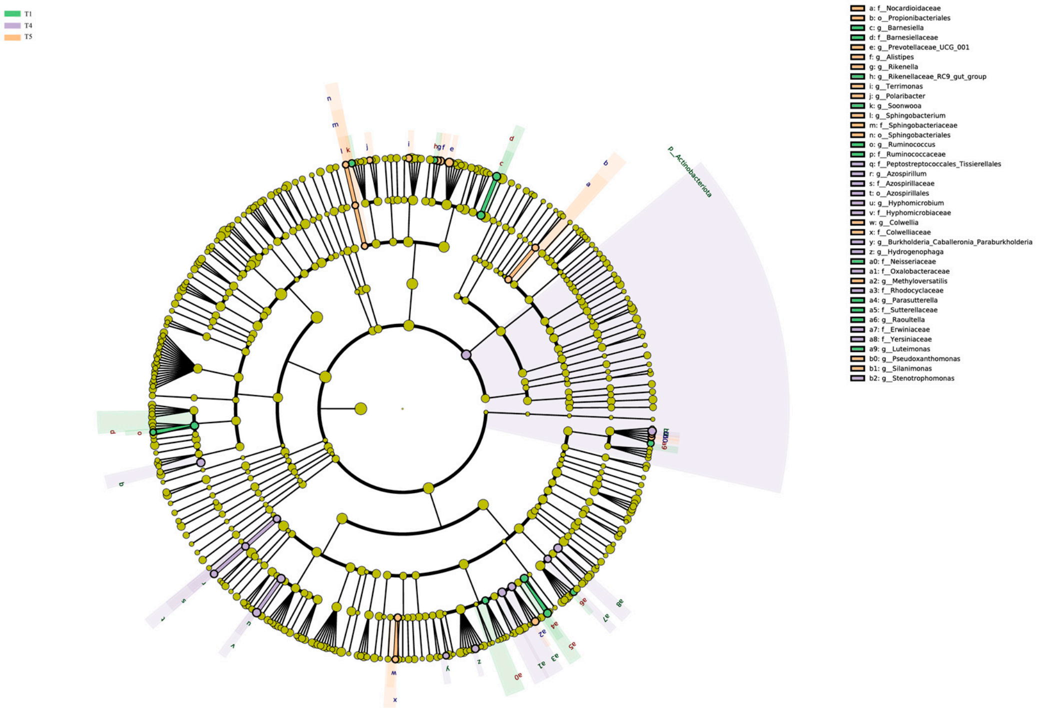
T1
T4
T5
p__Actinobacteriota
a: f__Nocardioidaceae
b: o__Propionibacteriales
c: g__Barnesiella
d: f__Barnesiellaceae
e: g__Prevotellaceae_UCG_001
f: g__Alistipes
g: g__Rikenella
h: g__Rikenellaceae_RC9_gut_group
i: g__Terrimonas
j: g__Polaribacter
k: g__Soonwooa
l: g__Sphingobacterium
m: f__Sphingobacteriaceae
n: o__Sphingobacteriales
o: g__Ruminococcus
p: f__Ruminococcaceae
q: f__Peptostreptococcales_Tissierellales
r: g__Azospirillum
s: f__Azospirillaceae
t: o__Azospirillales
u: g__Hyphomicrobium
v: f__Hyphomicrobiaceae
w: g__Colwellia
x: f__Colwelliaceae
y: g__Burkholderia_Caballeronia_Paraburkholderia
z: g__Hydrogenophaga
a0: f__Neisseriaceae
a1: f__Oxalobacteraceae
a2: g__Methyloversatilis
a3: f__Rhodocyclaceae
a4: g__Parasutterella
a5: f__Sutterellaceae
a6: g__Raoultella
a7: f__Erwiniaceae
a8: f__Yersiniaceae
a9: g__Luteimonas
b0: g__Pseudoxanthomonas
b1: g__Silanimonas
b2: g__Stenotrophomonas

(C)

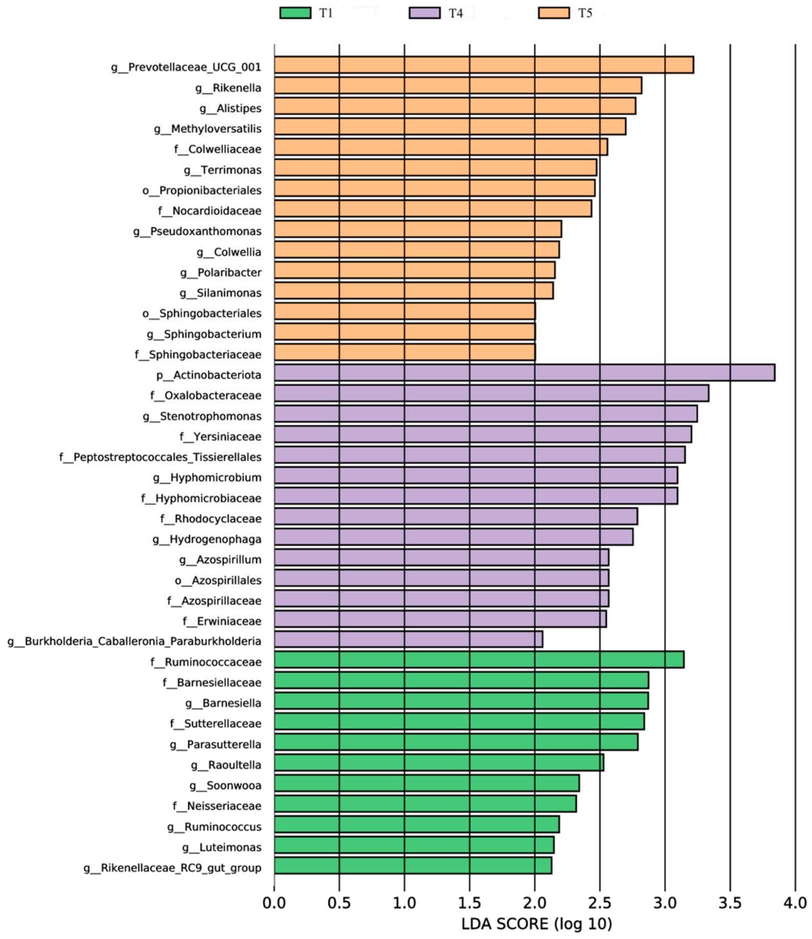
T1
T4
T5
g__Prevotellaceae_UCG_001
g__Rikenella
g__Alistipes
g__Methyloversatilis
f__Colwelliaceae
g__Terrimonas
o__Propionibacteriales
f__Nocardioidaceae
g__Pseudoxanthomonas
g__Colwellia
g__Polaribacter
g__Silanimonas
o__Sphingobacteriales
g__Sphingobacterium
f__Sphingobacteriaceae
p__Actinobacteriota
f__Oxalobacteraceae
g__Stenotrophomonas
f__Yersiniaceae
f__Peptostreptococcales_Tissierellales
g__Hyphomicrobium
f__Hyphomicrobiaceae
f__Rhodocyclaceae
g__Hydrogenophaga
g__Azospirillum
o__Azospirillales
f__Azospirillaceae
f__Erwiniaceae
g__Burkholderia_Caballeronia_Paraburkholderia
f__Ruminococcaceae
f__Barnesiellaceae
g__Barnesiella
f__Sutterellaceae
g__Parasutterella
g__Raoultella
g__Soonwooa
f__Neisseriaceae
g__Ruminococcus
g__Luteimonas
g__Rikenellaceae_RC9_gut_group
0.0
0.5
1.0
1.5
2.0
2.5
3.0
3.5
4.0
LDA SCORE (log 10)

(D)

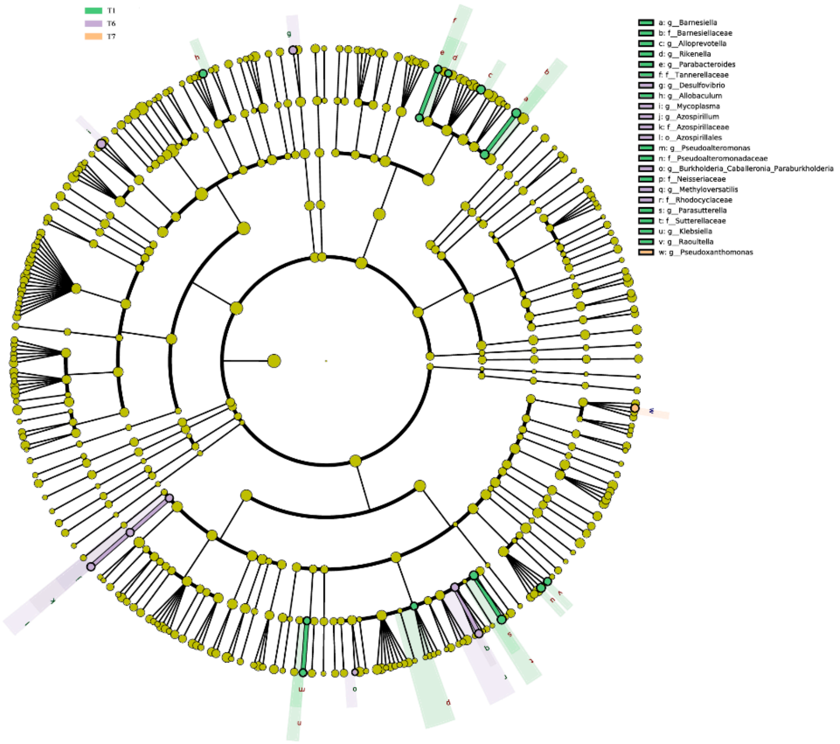
T1
T6
T7
a: g__Barnesiella
b: f__Barnesiellaceae
c: g__Alloprevotella
d: g__Rikenella
e: g__Parabacteroides
f: f__Tannerellaceae
g: g__Desulfovibrio
h: g__Allobaculum
i: g__Mycoplasma
j: g__Azospirillum
k: f__Azospirillaceae
l: o__Azospirillales
m: g__Pseudoalteromonas
n: f__Pseudoalteromonadaceae
o: g__Burkholderia_Caballeronia_Paraburkholderia
p: f__Neisseriaceae
q: g__Methyloversatilis
r: f__Rhodocyclaceae
s: g__Parasutterella
t: f__Sutterellaceae
u: g__Klebsiella
v: g__Raoultella
w: g__Pseudoxanthomonas

(E)

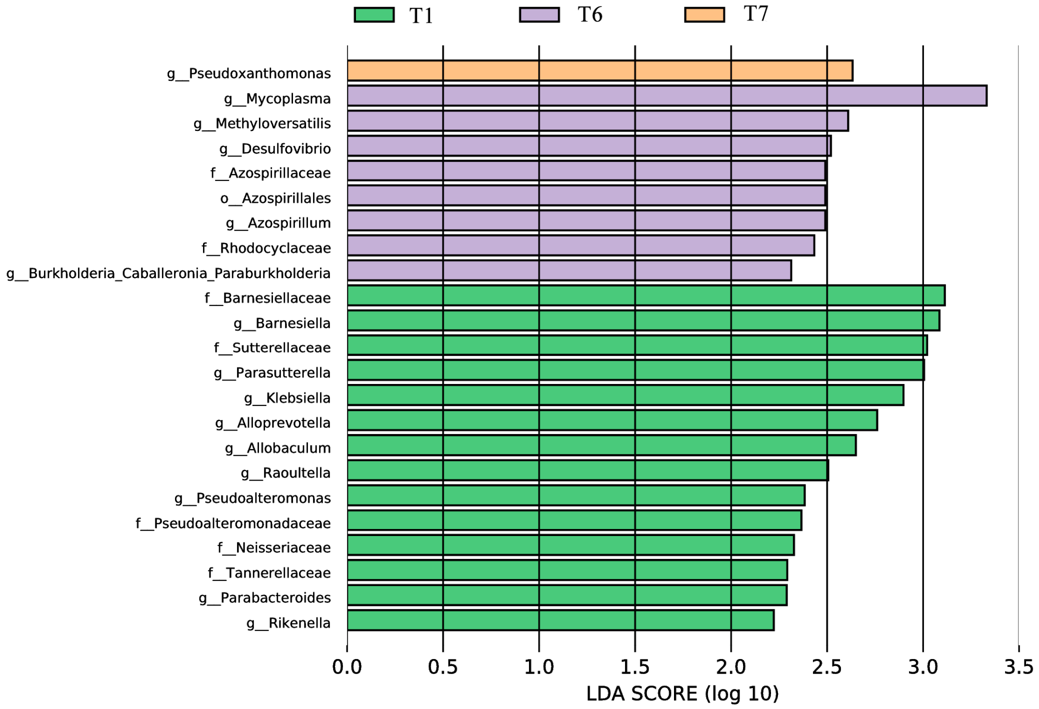
T1
T6
T7
g__Pseudoxanthomonas
g__Mycoplasma
g__Methyloversatilis
g__Desulfovibrio
f__Azospirillaceae
o__Azospirillales
g__Azospirillum
f__Rhodocyclaceae
g__Burkholderia_Caballeronia_Paraburkholderia
f__Barnesiellaceae
g__Barnesiella
f__Sutterellaceae
g__Parasutterella
g__Klebsiella
g__Alloprevotella
g__Allobaculum
g__Raoultella
g__Pseudoalteromonas
f__Pseudoalteromonadaceae
f__Neisseriaceae
f__Tannerellaceae
g__Parabacteroides
g__Rikenella
0.0
0.5
1.0
1.5
2.0
2.5
3.0
3.5
LDA SCORE (log 10)

(F)
